# Influence of homocysteine on regulating immunothrombosis: mechanisms and therapeutic potential in management of infections

**DOI:** 10.1007/s00011-025-02045-0

**Published:** 2025-05-24

**Authors:** Aswathy S Nair, Lloyd Tauro, Harshit B Joshi, Arnab Makhal, Teddy Sobczak, Julien Goret, Antoine Dewitte, Srinivas Kaveri, Harinath Chakrapani, Maria Mamani-Matsuda, Manjunath B Joshi

**Affiliations:** 1 Department of Ageing Research, Manipal School of Life Sciences, Manipal Academy of Higher Education, Manipal, Karnataka 576104 India; 2 Department of Chemistry, IISER, Pune, Maharashtra 411008 India; 3 CNRS, Immunoconcept, UMR 5164,Inserm ERL1303, Bordeaux University, 33000 Bordeaux, France; 4 Department of Immunology and Immunogenetics, Bordeaux University Hospital, 33000 Bordeaux, France; 5 Department of Anaesthesia and Intensive Care, Bordeaux University Hospital, F-33600 Pessac, France; 6 INSERM, Centre de Recherche des Cordeliers, F-75006 Paris, France

**Keywords:** Homocysteine, Immunothrombosis, Infection, Sepsis, COVID-19

## Abstract

Mechanisms controlling innate immune responses and coagulation are interdependent, evolutionarily entangled and make a complex network to form immuno-thrombosis axis which is an integral part of host-defence response. During infections, immunothrombosis generates intravascular scaffold enabling recognition, trap and destruction of pathogens facilitating tissue integrity. However, the accompanying dysregulation fosters into pathologies associated with thrombosis and regulates severity, morbidity and mortality in infections. Several extrinsic and intrinsic factors such as (epi)genetic mechanisms, age, metabolism and lifestyle regulate immunothrombosis during infections. Mounting evidence demonstrates that homocysteine, a metabolic intermediate of methionine synthesis pathway activate cells participating in immuno-thrombosis such as neutrophils, platelets, monocytes and endothelial cells. Interestingly, multiple infections are significantly associated with perturbed homocysteine metabolism. In the present review, we describe mechanistic insights into how homocysteine drives immuno-thrombotic crosstalk that generate a vicious cycle of inflammation and coagulation that fuels organ failure during infections with an emphasis on sepsis, COVID-19, and other infectious diseases caused by parasites, viral, and bacterial pathogens. Subsequently, we discuss therapeutic strategies targeting homocysteine metabolism that may improve clinical outcomes in infections.

## Introduction

Thrombosis is associated with pathogenesis of several diseases including infections, atherosclerosis, stroke, diabetes and cancers. Several extrinsic and intrinsic risk factors such as obesity, age, smoking, hyperglycemia, chronic inflammation, constitutive activation of coagulation factors and other clinical conditions trigger the thrombotic events in these diseases [1]. In vitro, pre-clinical and clinical studies have demonstrated complex and interdependent relationship between innate immunity, platelet activation and coagulation cascades leading to a state of immunothrombosis. ‘Immunothrombosis’ first coined by Engelmann and Massberg [2] is the process by which immune cells, such as platelets, neutrophils and monocytes along with endothelial cells, actively contribute to the development of thrombi within arteries, specifically in micro vessels. This is an essential host defence mechanism to maintain physiological homeostasis during infections or tissue damage to inhibit blood loss. However, abnormal and chronic activation of coagulation cascade results in disseminated intravascular coagulopathy (DIC) and other thrombotic processes have been observed in several pathologies including bacterial and viral infections, myocardial infarction, stroke, and venous thromboembolism [2–4]. Cardiovascular events often manifest in individuals with inflammatory diseases and during infections, notably in COVID-19, supports the therapeutic importance of aberrant immunothrombosis, also known as thromboinflammation. Exaggerated and unchecked immunothrombosis induces collateral damage, impairing organ functions through microvascular thrombosis.

Perturbed circulating metabolites in patients with thrombosis indicates underlying metabolic regulation and control over immunothrombosis. Hence, targeting these metabolic pathways offers the opportunity for developing anticoagulation therapies to avoid recurring events. Homocysteine, an intermediate formed in the methionine metabolism using cofactors such as vitamin B6, Vitamin B12 and folic acid actively participates in the methyl transfer of one-carbon metabolism and involved in physiological activities, such as redox defence, amino acid balance, epigenetic maintenance, and biosynthesis of nucleotides [5, 6]. Perturbed one carbon metabolism due to genetic determinants of methionine metabolism, vitamin deficiency, environmental-lifestyle-physiological factors and cellular signalling contribute to an elevation in the homocysteine levels termed hyperhomocysteinemia (HHcy). High plasma homocysteine levels are associated with the development and progression of multiple diseases with their pathophysiological role in endothelial dysfunction, inflammation, oxidative stress, and thrombosis [7–10].

The direct effect of homocysteine on inducing thrombosis in infections is less understood and however, high levels of homocysteine have been associated bacterial, viral and parasitic infections. Studies have shown higher plasma homocysteine and lower folate level in the *Helicobacter pylori* infection [11, 12]. Spontaneous bacterial peritonitis (SBP) showed elevated serum and ascitic homocysteine levels [13]. Virmani et al., 2018 demonstrated involvement of homocysteine in the biofilm formation by Mycobacteria [14]. Another case report indicated tuberculosis with hyperhomocysteinemia for the development of venous thromboembolism [15]. Increased homocysteine mediated oxidative stress cause complications in the pregnant women infected with Hepatitis E virus (HEV) [16]. In hepatitis C, activating the inhibitor of metalloproteinase TIMP-1(Tissue inhibitor of metalloproteinases-1) and decreasing the antiviral impact of interferon through hypomethylation of STAT1(Signal transducer and activator of transcription 1), hyperhomocysteinemia may contribute to hepatic fibrogenesis. In vitro studies indicated that normalising STAT1 methylation with betaine and S Adenosyl Methionine restore interferon’s antiviral activity [17]. HIV infected subjects with high homocysteine contribute thrombophilic abnormalities and cognitive impairment [18, 19]. Multiple studies showed the co-relation between increased serum homocysteine, elevated cytokine levels and decreased folate in COVID-19 positive individuals irrespective of age group and ethnicity [20–25]. Taken together, homocysteine levels may significantly regulate immnuothrombosis pathways and thereby influence severity and response of various infections.

In the present review we discuss (a) influence of homocysteine on cells participating in immunothrombosis; (b) perturbations of homocysteine metabolism in infections including sepsis and (c) therapeutic strategies to mitigate homocysteine metabolism in clinical management of infections.

## Interdependent activation of platelets, neutrophils and endothelial cells regulate immunothrombosis

Platelets and neutrophils mutually interact during the hemostasis and inflammation as a major contributor of vascular integrity. In the event of an aggressor, endothelial cells secrete molecules (laminin, fibrin, collagen, tissue factor) that promote the adhesion of platelets and neutrophils. Platelet activation leads to the interaction of GPIIb/IIIa with fibrinogen, improving the platelet-matrix extracellular matrix binding. Platelet secretion of CCL5 (chemokine C-C motif ligand 5), CXCL4 (platelet factor 4, PF4) and CXCL7 activates monocytes and neutrophils [26–28]. Platelets sense the exposed collagen in the subendothelial layer during vascular damage and react with them through von Willebrand factor (vWF) and glycoproteins. Further activated platelets secrete aggregatory mediators such as thromboxane, serotonin and ADP results in the thrombin formation and activation of GPIIb/IIIa integrins [29–31]. Exposure of P-selectin ligand on activated platelets enables the binding of non-activated neutrophils to them via P-selectin glycoprotein ligand-1 (PSGL1) and attract them to the site of thrombus formation. ɑ-granules secreted by platelets contain a set of chemokines such as platelet factor 4 (CXCL4) and the neutrophil activating peptide (CXCL7) which potently stimulate neutrophils [32, 33]. High mobility group box 1 (HMGB1) secreted by platelets activate the neutrophil receptors such as Toll like receptor-4 (TLR4) and receptor for advanced glycation end products (RAGE) to initiate the neutrophil extracellular trap formation. Addition to this, interleukin 1 beta (IL-1β), transforming growth factor beta (TGF-β) and cluster of differentiation 40 ligand (CD40L) also mediated the platelet dependent neutrophil activation [34, 35]. Neutrophil elastase and Cathepsin G released by the NETs promote proteolytic activation of receptors in platelets and potentiate the aggregation process. The neutrophil elastase also increases factor Xa activity and binds to factor XII to stimulates fibrin formation and protein C inhibition by histone through thrombomodulin accelerate the magnitude of fibrin generation [36, 37]. The histones possess high affinity to the membrane phospholipids and increase the calcium levels through ion influx results in the hyperactivation of endothelial cells. Monocyte-platelet aggregates (MPA) formed by the interaction of P-selectin/PSGL-1 leads to reactive oxygen species (ROS) production, NF-κB activation, release of molecules such as monocyte chemotactic protein-1 (MCP-1), tissue factor (TF), IL-1β and IL-8 and accelerate thrombosis [38, 39]. Elevated CRP levels increase expression of endothelial cell adhesion molecule, monocyte recruitment through monocyte tissue factor and leukocyte adhesion to the atherosclerotic plaque due to the reduced nitric oxide production in the endothelium [40]. Taken together, interdependent activation of platelets, endothelial cells and neutrophils intricately regulate immunothrombosis.

## Homocysteine robustly activate cell types participating in thrombosis

### Homocysteine activates p38 MAPKinase/cPLA_2_ in platelets

In vitro studies revealed that platelet aggregation and adhesion are boosted by homocysteine in the presence of agonists such as thrombin and collagen [41–43]. Higher local ADP concentrations brought on by Hcy-induced suppression of ADP hydrolysis may increase responsiveness to the agonists. It has been demonstrated that Hcy acts as an inducer of oxidative stress rather than directly affecting the enzymatic activity of apyrase (catalyzes the hydrolysis of ATP to yield AMP and pyrophosphate), and that the ROS produced under hyperhomocysteinemia are more likely the true modulators of ADP hydrolysis than the mechanism by which Hcy inhibits the hydrolysis of nucleotides [44]. Homocysteine also has the ability to raise the concentrations of a different platelet agonists: the acid arachidonate released from platelet membranes through a process mediated by A2 phospholipase in the cytosol (cPLA2). It has been demonstrated that Hcy modifies cPLA2’s phosphorylation status in an approach that is reliant on upstream mitogen-activated kinase p38 and calcium ions. These progressive modifications in the activation of signaling proteins lead to the enhanced release of arachidonic acid, leading to activation of platelets either auto- or paracrine manner. A second powerful platelet agonist, thromboxane A2, is produced by further metabolizing arachidonate. Reactive oxygen species are by-products of the metabolism of arachidonic acid. When arachidonic acid is being metabolized, cyclooxygenase 1, monooxygenase, and lipoxygenase can all play a significant role in synthesising reactive oxygen species [45–47]. In order to create a platelet plug via bridging fibrinogen, Hcy-dependent stimulation of platelet activation also necessitates the induction of the active form of GPIIb/IIIa integrin [48]. Hcy appears to have a major detrimental effect on the process involved in nitric oxide metabolism (NO), one of the primary inhibitors of blood platelets. Protein kinase C (PKC) and phospholipase C2 are required for Hcy’s inhibition of NO synthase activity, which reduces blood platelet NO generation. In parallel, the PKC-dependent mechanisms boost NADPH oxidase activity in homocysteine stimulated platelets [49]. Homocysteine inhibits thrombin-induced NO synthase activation and superoxide anion production. Therefore, it is likely that the presence or absence of other factors affecting platelet physiology, such as platelet agonists or antiplatelet medications, will have a significant impact on how Hcy affects blood platelets [50]. Mohan et al., 2009, showed a quick decline in basal platelet counts while administering homocysteine in a dose-dependent manner [41]. Furthermore, hyperhomocysteinemia suppresses platelet response to antiplatelet drugs due to their priming property to certain agonists [51].

### Homocysteine-mediates redox imbalance and increases expression of cell adhesion molecules to accelerate monocyte recruitment

**Fig. 1:**
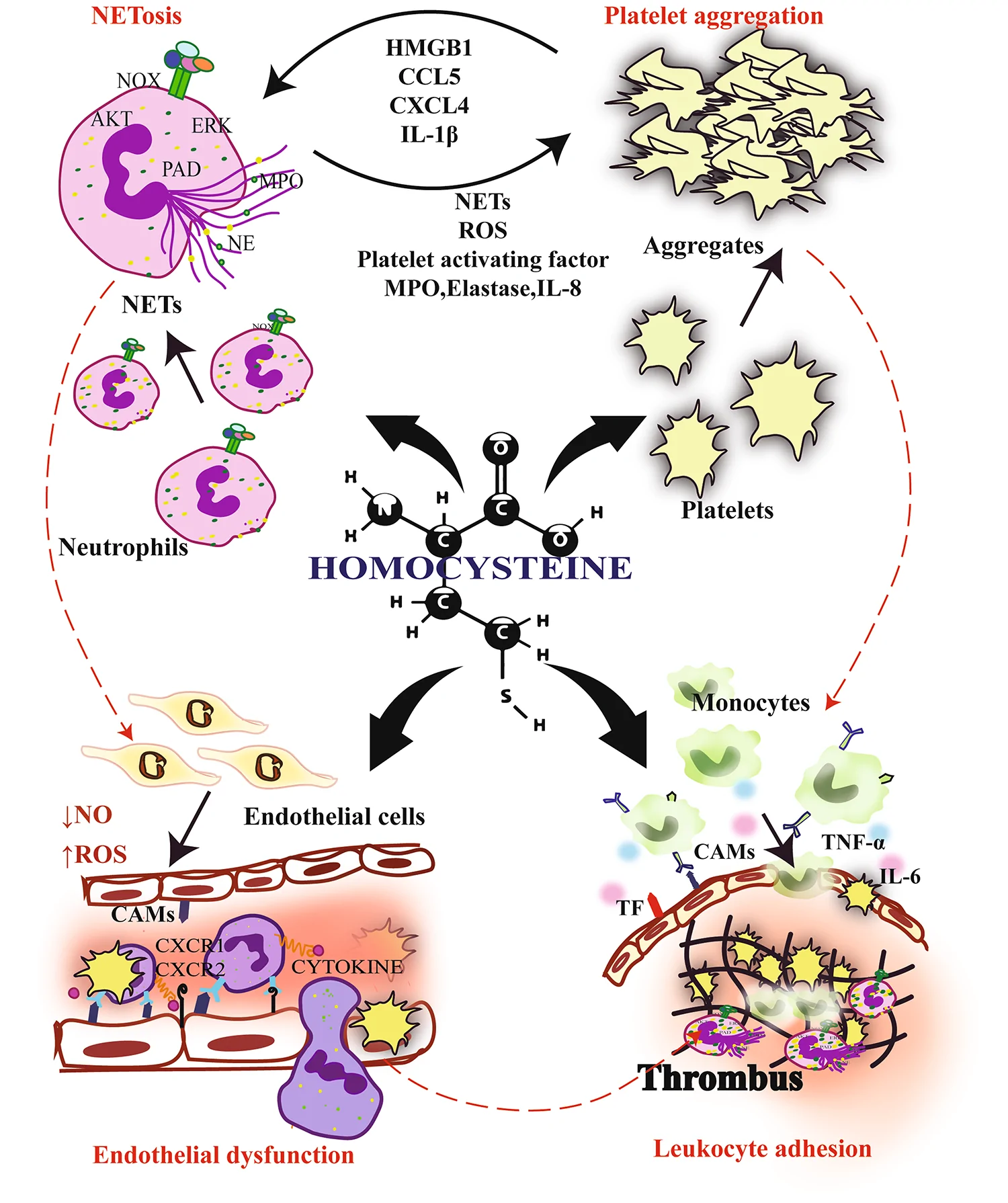
Proposed mechanism through which homocysteine activates the immune cells such as neutrophils, monocytes, platelets and endothelial cells to induce immunothrombosis. CAMs and TF represent cell adhesion molecules and tissue factor respectively

Koga et al., 2002 showed that homocysteine boosts expression of adhesion molecules to enhance endothelial cell interactions with T cells and monocytes, hence promoting the development of atherosclerosis. In the in-vitro study, homocysteine increased the leukocyte adhesion to endothelium derived cells while having no effect on other thiol-containing substances like cysteine. Homocysteine increased HAEC (Human Aortic Endothelial Cells) adherence to pro-monocytic human myeloid leukaemia cell line-U937 in concentrations of 20 µmol/l, which is considered as mild hyperhomocysteinemia. However, homocysteine increased U937 cell’s adhesion to HAEC in a dose-dependent way when treated to both HAEC and U937 cells. The significant rise in monocyte adhesion with Hcy as severe as it seen in profoundly hyperhomocyteinemic patients may point to higher incidence of atherogenesis in these individuals. In this study, authors showed that homocysteine increased E-selectin and VCAM-1 mRNA levels and stimulated endothelial cell surface expression [52]. Homocysteine increased the interaction between leukocytes and endothelial cells through adhesion molecules such as vascular monocyte adhesion-associated protein, connecting segment-1, fibronectin and P-selectin (Fig. 1) [53]. Endothelial cells treated with homocysteine generate more pro-inflammatory cytokines and chemokines, such as MCP-1 and IL-8, which draw leukocytes to the stimulation site and facilitates to transmigrate to the subendothelium [54, 55]. Redox imbalance is an early signal in the leukocyte adhesion response that sets off a cascade of molecular events. VCAM-1, E-selectin and ICAM-1 genes have binding sites for the NF-kB nuclear transcription factor, and ROS alter intracellular redox status, impact the genes that encode and activate their transcription factors and also affect the molecules involved in signal transduction. In cultured cells exposed to homocysteine, a change in redox status and reduced ROS in the presence of antioxidants were observed [56]. Homocysteine has also been demonstrated to activate MAP kinase, stimulate c-Myb and c-Fos, and induce c-Fos resulting in the production of adhesion molecules [57]. Reactive oxygen species, including hydrogen peroxide and superoxide, are produced during the auto-oxidation of homocysteine. ROS mediates inflammatory reactions either directly by boosting CAM expression or indirectly by inducing the production of cytokines like IL-1 [58]. Dorenkamp et al., 2021, demonstrated that monocytes increased chemokinesis and enhanced chemotaxis toward MCP-1 under hyperhomocysteine conditions. Additionally, when exposed to Hcy increased adhesion to inflamed endothelial cells under both physiological flow and static conditions demonstrates that monocytes have a pro-adhesive phenotype. Monocytes exposed to homocysteine showed decreased PTEN phosphatase (Phosphatase and tensin homolog) activity and function due to downregulation. The effect of homocysteine on the methylation-dependent inactivation PTEN was confirmed by azacitidine (AZA) based inhibition of DNMT1 and results in augmented adhesion and migration of monocytes. In line with that supplementation with cofactors (folic acid and vitamin B12) of Hcy metabolism also inhibits the Hcy-induced pro-adhesive and pro-migratory characteristics [59]. According to Xuewei et al., 2003, the upregulation of integrins CD11b/CD18 and CD14 due to hyperhomocysteinemia is important in the release of hydrogen peroxide, oxidase activity, transendothelial migration and monocyte adherence to endothelial cells [60].

### Elevated homocysteine induce bidirectional activation of neutrophils and platelets by excess NETs formation and platelet aggregation

Patients with essential hypertension (EH) and HHcy displayed higher plasma MPO-DNA (myeloperoxidase DNA) and cf-DNA (cell-free DNA) levels than patients with EH alone explained the effective NETs formation in hyperhomocysteinemia. Homocysteine induces the release of neutrophil extracellular traps in Ca^2+^/ERK1/2/PAD4 dependent manner. Cleavage of NETs by DNase 1 in the EH and HHcy patients inhibits the procoagulant action demonstrating the crucial role of NETs in controlling the hypercoagulable condition in these patients. NETs extracted from these subjects displayed strong cytotoxic effects on ECs (endothelial cells) via ER stress, elevating CHOP, Caspase-12 and GRP78 expression. Previous studies have shown that cell-free histones activate platelets, which causes an increase in thrombin [61, 62]. Martos et al., 2020 showed significant levels of neutrophil markers (cell free DNA, MPO and calprotectin) and lower level of natural anticoagulant activated protein C (APC) and its genetic regulators (*THBD*-c.1418 T and, *PROCR*-H3, *PROCR*-H1) in patients with venous thromboembolism [63]. Earlier in vitro studies showed the inhibitory action on the activation of protein C by homocysteine suggesting an atherogenic effect on the venous thromboembolism [64, 65]. Another study with a hyperhomocysteinemia rat model showed the inhibitory effect of H_2_S on ROS formation and NETs production by arresting the platelet and endothelial cell activation [66]. Earlier findings from our lab demonstrated significant elevation of homocysteine in diabetic subjects causes the release of NETs via Ca^2+/^PAD4 and induces platelet aggregation (Fig. 1) [67].

### Homocysteine reduces nitric oxide availability in endothelial cells

Homocysteine impairs the transport of L-arginine, the precursor of NO into endothelial cells and increases the production of ROS by NADPH oxidase, which may cause endothelial nitric oxide synthase (e-NOS) uncoupling. Reduced NO bioavailability and inflammation are the results of the vicious cycle of additional e-NOS uncoupling and destruction (Fig. 1). Additionally, homocysteine causes the e-NOS inhibitor ADMA to accumulate and the degradation of the enzyme Dimethylarginine dimethylaminohydrolase (DDAH) [68]. Homocysteine produces homocysteine thiolactone in a different process, which targets lysine-rich proteins and may cause an endothelial apoptotic response in response to ER stress. The stress response genes GRP78 and GADD153, regulating thiol metabolism are elevated in response to homocysteine [69]. An in vitro study with aortic endothelial cells treated with Hcy in a concentration dependent manner demonstrated reduced antithrombin III binding activity [70]. Lentz et al., 1996 using a diet induced HHcy reported significant decrease in thrombomodulin activity [71]. In addition to this homocysteinuria patients also reported to have lower antithrombin III levels [72]. HHcy enhances the binding of lipoprotein to fibrin and reduces the binding of tissue plasminogen activator to endothelial cells causing diminished fibrinolytic activity [73, 74]. HUVECs treated with homocysteine in various concentration showed externalization of phophatidylserine and microparticles involved in clot promoting activity. Authors also observed that blocking of phosphatidylserine with annexin reduced 70% of clot formation and microparticles [75].

## Immunothrombosis in infectious diseases and constructing a relationship with Hyperhomocysteinemia

### Immunothrombosis and parasitic infections: example of malaria

Cerebral involvement leads to significant morbidity and mortality in subjects with malaria infection [76]. Accumulation of platelets in the cerebral microcirculation has been observed in children who died from cerebral involvement [77]. The cytoadherence of infected red blood cells (known as “knobs”) is recognized as a major factor in the vascular complications of malaria. *P. falciparum* erythrocyte membrane protein-1 (PfEMP-1), which is expressed by knobs, facilitate the cytoadherence to the vascular wall. Infected red blood cells are recognized by platelets, activating them. P-selectin and tissue factor are markers of platelet activation observed [78]. Platelet activation, their binding to von Willebrand factor, and vascular damage caused by infected red blood cells lead to a state of hypercoagulability [79]. Furthermore, there is an increase in procoagulant proteins (thrombomodulin, von Willebrand factor) and a decrease in anticoagulant proteins (protein C, antithrombin) favoring hypercoagulability and immunothrombosis [80, 81]. Von Willebrand polymers promote the adhesion of infected red blood cells to the vascular wall. Platelets form aggregates with infected red blood cells via CD36 and with leukocytes via the CD40-CD40L pair, promoting vascular sequestration of red blood cells and immunothrombosis in the microcirculation, particularly in the cerebral microcirculation. An in vitro blood coagulation test performed on the subjects first detected with *P. falciparum* parasitaemia displayed a higher thrombin generation potential (17.7%) [82]. Similarly, a case report with two cases of strongyloidiasis, another parasitic infection caused by *Strongyloides stercoralis*, displayed hypercoagulability and concurrent eosinophilia leads to thrombotic events [83]. Studies in parasite infections show an increase of homocysteine as consequence of parasite metabolism that could be used as predictive marker of severity, as proposed for *Plasmodium falciparum* [84]. Another study including the in vitro and in vivo model of malaria confirmed how higher levels of homocysteine triggers gametocytogenesis due to disrupted transsulphuration pathway [85].

### Immunothrombosis and bacterial infections

a) Gram-negative bacterial infections.

LPS, a component of Gram-negative bacteria, binds to TLR-2 and − 4. This interaction increases intracellular GMP and levels of activated protein kinase G. TLR-4 induces the activation of MYD88/NF-κB and MAP kinase pathways [86]. Recognition of LPS by platelets promotes their adhesion to the endothelium via P-selectin and GPIb and their production of molecules promoting hypercoagulability and immunothrombosis (tissue factor, CD40L, TNF-alpha, and PF4). The recognition of LPS by TLR-4, induces also platelet secretion of IL-1β through the NF-kB pathway, increasing the activation of endothelial cells and leading to vascular inflammation. Clarke et al., 2007 identified that TLR4 on platelets causes thrombocytes to bind to and activate neutrophils to release NETs while stimulation with exogenous LPS and under flow conditions NETs formed in the sinusoidal capillaries trapped the bacteria in the vasculature [62]. Molecules released by neutrophils (histones, serine proteases) activate platelets, amplifying immunothrombosis [61]. For example, histones, by binding to P-selectin and TLR 2/4, activate and aggregate platelets and induce platelets-mediated thrombin formation [87]. Data obtained from the human infective endocarditis revealed massive recruit of leukocytes and the presence of proteases such as elastase, MPO in the septic thrombi present in the valves and proteolytic aggregation as a result of bacterial colonization [88]. Multiple studies with in vivo model have shown how the caspase pathway get activated upon recognition of LPS (triggered by HMGB1) followed by pyroptois results in tissue factor driven immunothrombosis [89, 90]. Β1-defensins and HMGB-1 also induce NETosis. P-selectin and CD40L promote platelet-leukocyte interaction and amplify NETosis, contributing to immunothrombosis [91]. Hitchcock et al., 2015 showed inflammation driven thrombosis via ligation of C-type lectin–like receptor-2 (CLEC-2) on platelets using a mice model of *Salmonella* *typhimurium* infection [92]. In addition to these mice infected with *Salmonella typhimurium* shows up early onset of immunothrombosis in spleen (24 h) but a later response in liver despite of comparable bacterial burden in these organs suggesting the pathogenic role of immunothrombosis in systemic infection than accelerating bacterial containment [93]. A rodent model of streptococcal sepsis supported the functional contribution of contact system on bacterial dissemination and increased deposition of intravascular tissue factor in kidney [94].

b) Gram-positive bacterial infections.

*Staphyococcus aureus* is a Gram-positive coccus responsible for bacteremia, septic shock, and/or toxin shock. Platelets directly bind to *S. aureus* through multiple receptors (such as gC1qR) [95]. *S. aureus* secretes several virulence factors such as alpha toxin or SSL5 (Staphylococcal superantigen-like 5). The alpha toxin forms heptamers causing pores in cells and inducing their death [96]. The alpha toxin induces platelet activation and the release of antimicrobial peptides such as tPMP-1 (thrombin-induced platelet microbiocidal protein-1) and human β-defensin-1 [97]. These promote NETosis and contribute to the phenomenon of immunothrombosis. GPIbα and integrin αIIbβ3, by interacting respectively with integrin αMβ2 and SLC44 A2 (solute carrier family 44 member 2) on neutrophils, are also important players in NETosis [87, 98]. NETosis trigger the autoactivation of factor XII, contributing to immunothrombosis. Furthermore, NETosis citrullinate and inactivate ADAMTS13, a metalloprotease degrading vWF polymers, promoting a prothrombotic environment [99] vWF, via platelet GPIbα and neutrophil αMβ2, maintains the PNN-platelet interaction and enhance NETosis [100]. *S. aureus* triggers the formation of fibrin by activating prothrombin through the coagulases von Willebrand factor binding protein (vWbp) and staphylocoagulase (Coa) [101]. In *S. aureus* infected-mice, NETosis promote immunothrombosis by vWF binding to microvasculature and lead to liver injury. The intravenous injection of alpha toxin quickly leads to platelet recruitment, aggregation, and thrombus formation in hepatic sinusoidal capillaries and renal glomerular capillaries, contributing to organ failure in mice [102]. Neutrophil extracellular traps were detected in the fibrin sheaths from dialysis patients with a predominance of *Staphylococcus aureus* (69%). In the same study treatment with DNase I significantly reduces fibrin sheath and NET formation in rat model of transient bacteremia suggesting prominent effect of immunothrombosis in catheter-related central venous stenosis [94]. *S. aureus* stabilizes platelet adhesion with Kupffer cells, allowing for increased recruitment of neutrophils in hepatic sinusoidal capillaries and contributing to liver injury [103]. Integrin αIIbβ3 leads to platelet aggregation and increases platelet production of Bcl3 promoting the stabilization of intravascular thrombi and actively participating in immunothrombosis [104]. SSL-5 binds to platelet GPVI or GPIbalpha leading to the activation of integrin alphaIIb beta3 and the expression of P-selectin [105].

Recent studies have explained underlying mechanisms of inflammasome- and STING-driven immunothrombosis induced by bacterial infections and how targeting these pathways along with canonical (NLRP3) or noncanonical (caspase-11) pathways can block immunothrombotic events [106]. A study by Zhang et al., 2020 demonstrated the STING dependent coagulation pathway activation in association with calcium mediated Gasdermin D (GSDMD) and the improval of bacteremia with *Escherichia coli* and *Streptococcus pneumoniae* infection while inhibiting the STING-GSDMD pathway using using pharmacological inhibitors [107]. Human whole-blood model of *S. aureus* bacteremia and *E.coli* induced platelet aggregation emphasized the compliment mediated coagulation events and the cross talk with thromboinflammation [108, 109].

Statistical associations between homocysteine and bacterial infections have been widely reported. In bacterial pneumonia, elevated homocysteine correlated with poor outcome [110], as in vaginosis, homocysteine was associated with prevalence [111]. Homocysteine was proposed as diagnosis marker in bacterial peritonitis [13]. In bacterial -but not viral- meningitis homocysteine accumulates in the central nervous systems of patients [112]. Depending on bacteria and cohort population, such association is not constant. In *H. pylori* infection, homocysteine was only negatively correlated with seropositivity in Mexican Americans [113]. A clinical study, which included 101 patients with infected (IVU) and noninfected venous leg ulcers (NVU), discovered that IVUs had greater levels of hyperhomocysteinemia and inflammatory cytokines (IL-6, IL-10, IL17 A, and TNF-α), as well as substantial increases in wound size and depth. Strong biofilm producers were significantly more prevalent in IVUs, and Gram-negative bacteria made up 51.7% of the bacteria, compared to 48.3% of Gram-positive bacteria [114].

### Immunothrombosis in sepsis and possible relationship with hyperhomocysteinemia

**Fig. 2:**
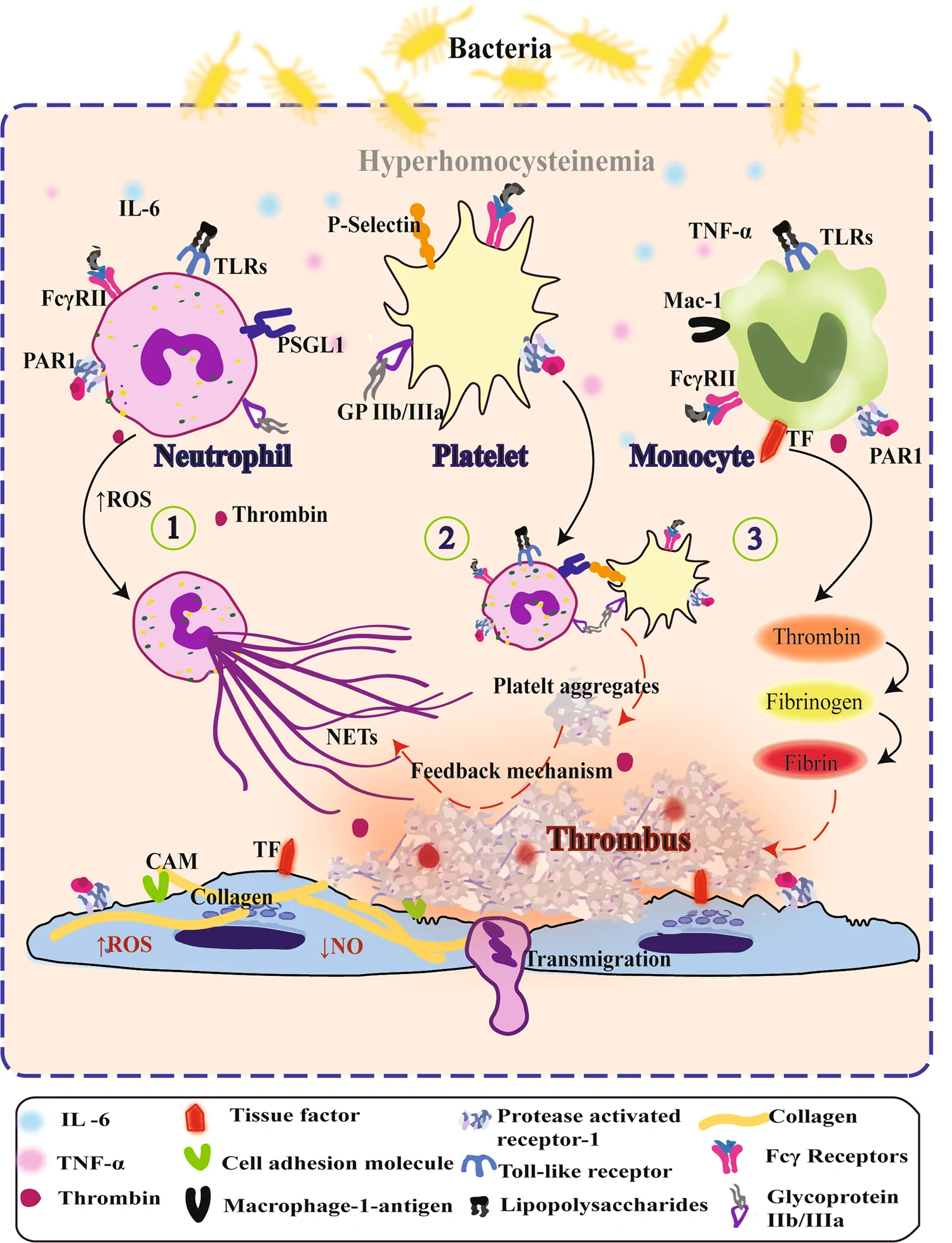
Hyperhomocysteinemia induced immunothrombosis in sepsis. Hyperhomocysteinemia causes oxidative stress and endothelial dysfunction by increasing tissue factor (TF) expression in endothelial cells and monocytes. Pro-inflammatory cytokines are released by activated endothelial cells and monocytes, which promotes further activation of immune cells 1) Activated neutrophils release extracellular traps. 2) PSGL-1 on neutrophils interacts with the p-selectin present in activated platelets and causes platelet aggregation and neutrophil transmigration. 3)Tissue factor binds to factor VIIa (TF-FVIIa), activating the extrinsic coagulation pathway and generating thrombin. Thrombin turns fibrinogen into fibrin, which forms a clot. These processes all contribute to a hypercoagulable condition and the development of thrombosis in sepsis

Sepsis is defined as life-threatening organ dysfunction caused by a dysregulated host response to infection. Despite recent advances in medicine, sepsis remains one of the leading causes of death in critically ill patients with a high rate of mortality up to 30% [115]. The microcirculation plays a key role in the development of multiple organ dysfunctions and is particularly vulnerable to the interactions between inflammation, coagulation and innate immune activation (Fig. 2). Neutrophils are the primary effector cells of innate immunity during sepsis. In certain conditions, according to pathogen’s characteristics, neutrophils release NETs which bind, kill and prevent further spread of the pathogens. Although NETs have protective roles at the first steps of infection, NETosis also seems to be dysregulated in sepsis patients. An excessive circulating ecDNA, nucleosomes and citrullinated-histone (Cit-H3) amount in blood induce thrombosis, intravascular coagulation and multiple organ failure in murine sepsis models. Concentration of extracellular DNA (ecDNA) in plasma of septic patients is higher in comparison to healthy controls and is associated with worse prognosis in ICU. The innate immunity and inflammation are implicated in the initiation and regulation of NETosis and release of extracellular DNA (ecDNA) [116]. Platelet-neutrophil interactions are broadly divided into 2 major pathways: secretion of soluble mediators (platelet factor 4–PF4-, CD40L, CXCL4 and CCL5, beta thromboglobulin, platelet microparticles, soluble CD62, RANTES, GRO-alpha and HMGB-1) and expression/activation of adhesion molecules, notably, platelets overexpress P-selectin that activate NETosis [62, 117]. However, the effects of chronic antiplatelet therapy on the severity of illness and outcomes are controversial in clinical studies [118].

In sepsis, different studies reported similar rates of plasmatic homocysteine in patients, compared to non-sepsis patients [119, 120]. Similarly, homocysteine levels were not correlated to SOFA score in a cohort of severe sepsis or septic shock patients with less than 48 h from organ dysfunction start, neither was the evolution of the homocysteine levels in the 3 first days following a septic shock [121]. When specifically looking at ventilated patients, homocysteine was not a significant parameter of prognosis [122]. A case-control cohort consisting of 109 sepsis subjects analysed for the methionine metabolites such as SAM, SAH, methionine and total homocysteine showed abnormal concentration and linked clinical outcomes [123]. Erdem et al., 2012 investigated homocysteine level in the patients with severe sepsis along with serum ischemia-modified albumin (IMA), malondialdehyde (MDA), folic acid and vitamin B (12), and their association with high-sensitivity C-reactive protein compared to healthy controls. Significantly higher serum IMA, homocysteine and MDA levels were observed in the severe sepsis with positive correlation with high-sensitivity C-reactive protein [124].

Nevertheless, methionine metabolism in sepsis is perturbated. SAM and SAH levels were shown higher in sepsis patients. S-adenosylhomocysteine (SAH) but not lactate, taken upon patient’s inclusion in the study close to ICU admission, significantly and independently contributed to the prediction of disease progression and death [119] and SAM/SAH ratio was shown increased in a sepsis model in rats [125] as well as in human patients [120, 123]. In such patients, homocysteine levels were unchanged or decreased as compared to healthy patients. High levels of homocysteine are described in elderly [126], whereas lower Hcy levels were shown in sepsis patients of the same aged cohort, correlating with high neutrophil numbers and decreased serum albumin. Yet, an early increase (< 24 h), of homocysteine has been associated with poor sepsis outcome, as shown by Ploder et al., 2010 [122]. Polymorphism in the cystathionine beta-synthase variable number tandemrepeats were associated with susceptibility to severe sepsis or septic shock which contribute the elevated homocysteine levels demonstrated by independent studies [127].

### Immunothrombosis and viral infections

a) Example of Dengue.

In every 10% of Dengue fever cases, thrombocytopenia and a hemorrhagic syndrome can be observed (DHF - dengue hemorrhagic fever), which can progress to dengue hemorrhagic shock (DHS). DENV (Dengue virus) can directly bind to platelets through several receptors such as DC-SIGN; HSPG; TLR2; TLR2 and CLEC-2 leading to their activation. Platelets are also capable of recognizing dengue viruses opsonized by Ig via Fc receptors [128]. This recognition leads to the degranulation of platelet contents, including serotonin, which appears to contribute to the development of DHS and is known to enhance thrombocytopenia [129]. Platelet activation by DENV is associated with an increase in NLRP3 inflammasome formation, which by activating caspase-1, promotes platelet production of IL-1 β, platelet aggregation, and platelet apoptosis [130]. Inhibition of caspase-1 and IL-1 β reduces hemorrhagic lesions and increases platelet count in DENV-infected mice [129]. Platelet-monocyte interaction (via the P-selectin/PSGL1 couple, for example) leads to platelet and monocyte production of IL-1 β and IL-8 [131]. These cytokines are responsible for increased vascular permeability. After activation, platelets undergo apoptosis and leads to coagulation activation, thrombin generation, and thrombus formation. Platelet activation and apoptosis are associated with the severity of thrombocytopenia and are more prominent in patients with DHF [128].

b) Example of COVID-19 and possible relationship with hyperhomocysteinemia.

**Fig. 3:**
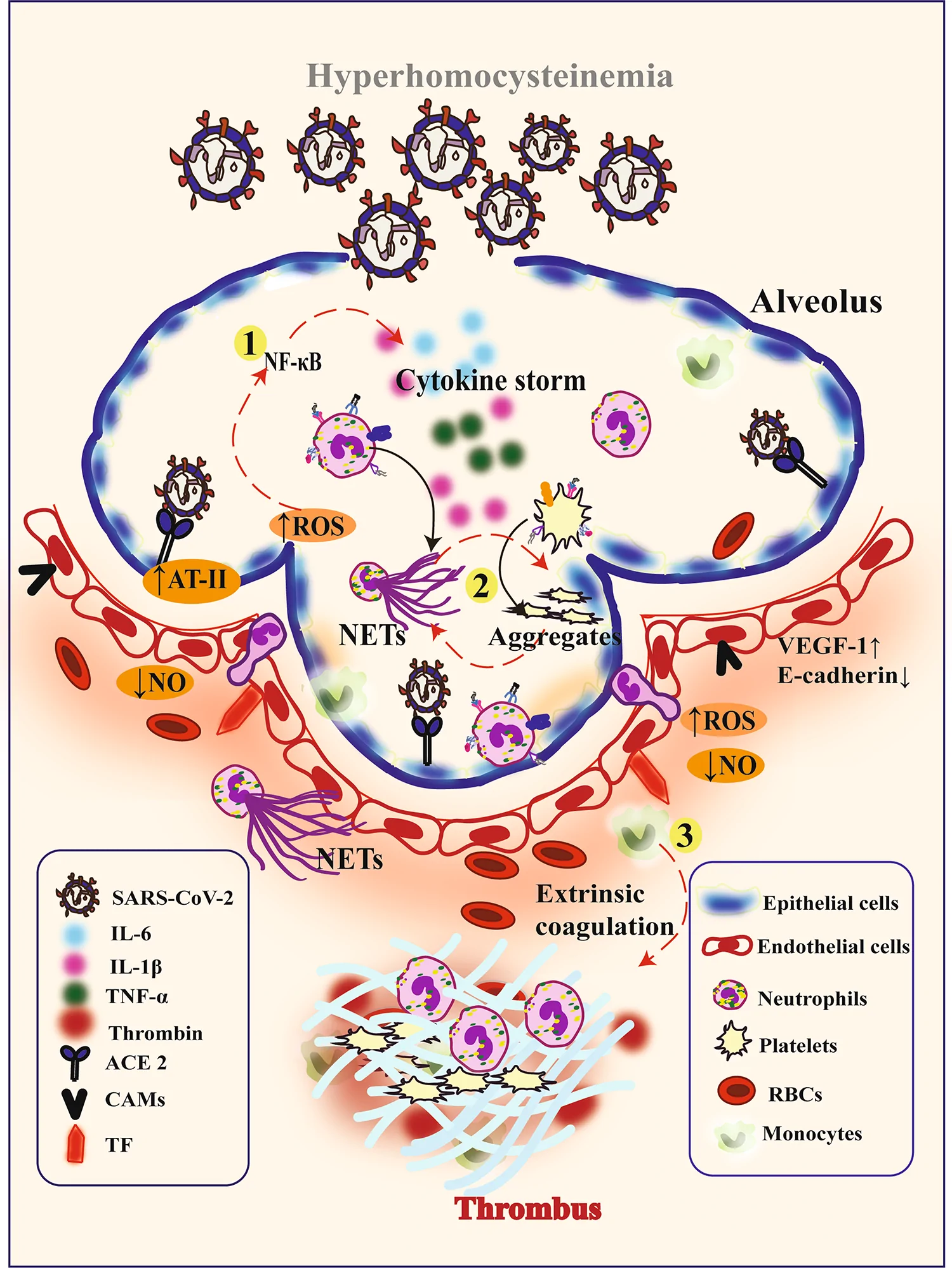
Hyperhomocysteinemia induced immunothrombosis in COVID-19. Hyperhomocysteinemia causes reduced ACE2 expression and increased angiotensin 2 levels. 1) Oxidative stress associated with this accelerates cytokine production through the NF-κB pathway resulting in a “Cytokine storm”. 2) The cytokines stimulate the neutrophils and platelets for feedback loop interactions. 3) Angiotensin II directly induces the tissue factor expression on monocytes and initiates the extrinsic coagulation cascade and the fibrin formation. Reduced NO availability and increased ROS in the endothelial cells lead to constant inflammation and promote thrombogenic events

During inflammatory responses, platelet-neutrophil interactions are significantly elevated. These interactions are initiated by soluble mediators, which then directly activate these cells. Platelet adhesion to neutrophils when infected patient’s plasma was co-cultured with neutrophils and platelets were mediated by the TLR4 receptor [132]. Selectin and integrin family adhesion molecules are expressed by activated neutrophils to facilitate the interaction of platelets and endothelium [133]. The single stranded RNA virus SARS-CoV-2 has recently been shown to increase platelet function and promote the development of platelet-monocyte complexes through Tissue Factor expression of monocytes (Fig. 3). Platelet-SARS-CoV-2 interaction encourages apoptosis in platelets. Immunofluorescence, transmission electron microscopy, and RNA sequence analysis for SARS-CoV-2 indicated by ARTIC v3 sequencing have shown that SARS-CoV-2 virions internalised when attached to microscopic particles [134]. Such internalisation leads to necroptosis, apoptosis, and EV release, and affects immunity with increased risk of thrombosis. According to several studies, platelets may bind to SARSCoV-2 RNA molecules, and older patients could experience this occurrence. It is possible to learn more about arterial thrombosis from a metabolite or biomolecule linked to active platelets. The biomarkers include platelet-derived extracellular vesicles (PEV), which include exosomes and platelet microparticles or micro vesicles. The tiny vesicles known as platelet-derived extracellular vesicles (PEVs) are generated from activated platelets and are presumably the mechanism by which several advantages attributed to platelets are exerted. The platelet cytomembrane component’s extrusion causes the vesicle release with the help of the platelet plasma membrane. Small PEVs with a diameter of 80–200 nm and larger PEVs with a diameter of 400–600 nm are confirmed by electron microscopy. These vesicles nevertheless exhibited procoagulant activity that was mediated by tissue factor and factor V-like activity. Extracellular vesicles produced by platelets can act as indicators for autoimmune disorders, cancer, cardiovascular conditions, and infections. Activated platelets and neutrophils attract one another to inflammatory or wounded tissues, which results in thrombo-inflammation [135]. Plasma homocysteine levels, monocyte lymphocyte ratio, and age of the patients were examined in a study of individuals who had been diagnosed with COVID-19. The homocysteine values were 9.3 ± 0.2 mM in the group with moderate disease and 10.7 ± 0.5 mM in the group with severe disease suggesting the predictive levels of homocysteine with the disease severity [21]. Additionally, it has been reported that the disease’s progression causes the monocyte lymphocyte ratio to rise dramatically. Thrombotic disease, enhanced fibrinolysis, and intravascular coagulation may all be indicated by elevated D-dimer levels. The cytokine storm, tissue injury, or maybe the development of sepsis is indicated by the elevated D-dimer, which can be observed in the severe cases of COVID-19. When measured alongside D-dimer, monocyte lymphocyte ratio and homocysteine may provide better insights about the COVID-19 severity in patients. In COVID-19 patients, high homocysteine can be viewed as a risk factor. One may say that people with hyperhomocysteinemia are at risk for COVID-19 illness [21].

## Therapeutic strategies to ameliorate immuno-thrombosis due to homocysteine

Hyperhomocysteinemia or the dysregulated homocysteine metabolism is currently considered as the therapeutic target rather than its diagnostic nature in the pathological condition with vitamin supplementation being a major focus. Supplementation of folic acid, vitamin B6&B12 and betaine reduces thrombotic and cardiovascular events in subjects with HHcy. In addition to that folic acid administration determines the platelet activation and degree of lipid peroxidation [136]. Daily supplementation of folic acid lowers plasma Hcy level by 25% and a combination of folic acid and vitamin B12 additionally lowers the level by 7% indicating the critical role of these vitamin B supplements in the regulation of homocysteine metabolism [137]. Furthermore, dietary management of homocysteine has also been studied extensively. An inverse association of whole-grain intake and Hcy was observed irrespective of gender with a mean reduction 17% plasma homocysteine levels. Interestingly, the mediterranean diet and fiber-rich diet also showed a decline in Hcy levels within a few weeks of administration [138]. Increased intake of vegetables and fruits rich in phytochemicals exerts health benefits in inflammatory conditions and endothelial dysfunction due to their antioxidant properties [139]. Cumulative data suggests various studies with genistein, epigallocatechin-3-gallate (EGCG), curcumin and quercetin reduce the plasma homocysteine levels and regulate the oxidative stress, eNOS expression and enzymes associated with the antioxidant system to prevent homocysteine induced damage. Nutritional recommendations for the subjects with genetic mutations follow alternative strategy to maintain normal Hcy levels. A low methionine diet is beneficial for subjects with SAH hydrolase, Met adenosyltransferase deficiency, whereas a low methionine diet with betaine is recommended for patients with CBS deficiency [140]. Pre-clinical testing of a novel enzyme therapy using OT-58 (Modified human CBS) showed its potential efficacy in lowering plasma homocysteine in the clinical symptoms of homocysteineuria [141].

**Fig. 4:**
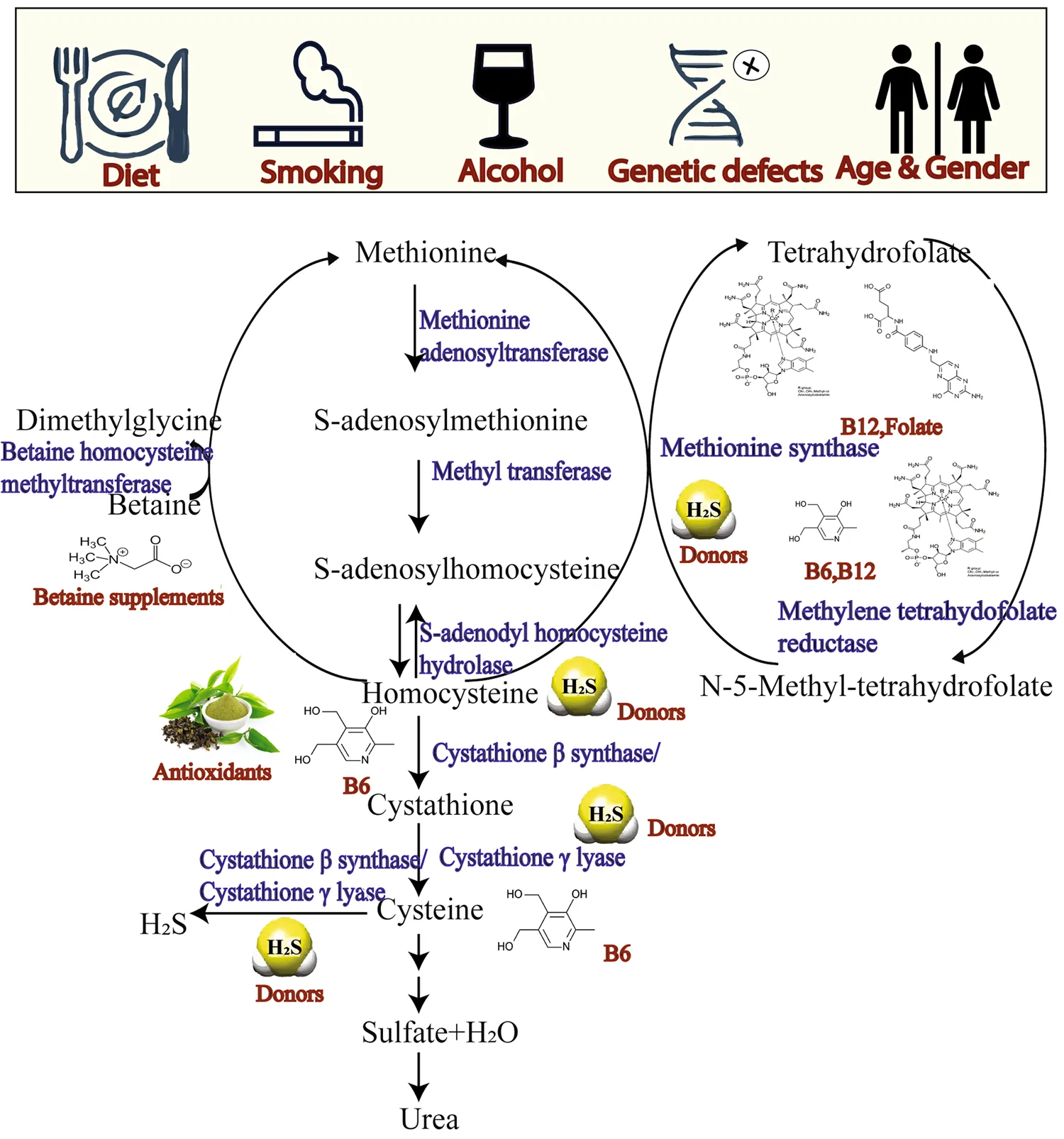
Homocysteine metabolism and therapeutic approaches to reduce hyperhomocysteinemia

The cellular levels of hydrogen sulfide (H_2_S) and related species are linked to homocysteine’s levels and effects [140]. Hydrogen sulfide (H_2_S) is an important mediator of antioxidant response, and acts through multiple mechanisms [142]. In cells, cystathionine β-synthase, cystathionine γ-lyase (CSE), cysteinyl-tRNA synthetase (CARS) and 3-mercaptopyruvate sulfurtransferase (3-MST) contribute to H_2_S biosynthesis (Fig. 4) [143, 144]. Among these enzymes, CBS and CSE are mainly located in the cytosol, and under certain conditions can translocate to mitochondria [145]. CARS is known to be present in both cytosol (CARS1) as well as mitochondria (CARS2) [146, 147]. On the other hand, the expression levels of 3-MST is 2.5–3-fold higher in mitochondria compared to the cytosol and significantly contributes to H_2_S biosynthesis in mitochondria [148]. The ultimate source of H_2_S in cells through enzymatic biosynthesis is cysteine. The uptake of cystine in cells, followed by reduction of cystine produces cysteine, which is used as is, or is converted to 3-mercaptopyruvate, which is in turn a substrate for 3-MST [149] Collectively, these pathways work to maintain levels of H_2_S in cells, and are reflective of overall cellular health. Protein persulfidation, a post-translational modification has recently been found to mediate many of the effects of H_2_S [150, 151] Methionine demethylation yields homocysteine (Hcy), a sulfur-containing nonessential amino acid that functions as an intermediate metabolic product. Two protons (H^+^), two electrons (e^−^), and the oxidized disulfide (homocysteine) are produced when two homocysteine molecules oxidize, along with the creation of reactive oxygen species (ROS) [152].

Homocysteine derived from methionine promotes neurological disorders through endothelial cell dysfunction. In mouse brain endothelial cells (bEnd3), methionine treatment increased NADPH-oxidase-4 (NOX-4) expression and mitigated thioredoxin-1 (Trx-1) expression, thus leading to an increase in ROS level. Pretreatment of bEnd3 cells with an exogenous H_2_S donor, NaSH inhibits ROS production in the presence of methionine and protects them from oxidative stress [153]. When homocysteine was injected intracerebrally into rats, it increased malondialdehyde, nitrite levels and decreased glutathione levels in rats, causing oxidative-nitrosative stress. Intracerebral administration of NaSH attenuates oxidative stress [154]. Nath et al., 2019 have also shown oral administration of homocysteine increases superoxide and nitrite levels with a concomitant decrease in endogenous H_2_S levels. Oral supplementation of NaSH lessened homocysteine-induced ROS generation and neurodegeneration [155]. Oxidative stress induced by homocysteine can activate matrix metalloprotease (MMP), which leads to inflammatory processes such as atherosclerosis [156]. Vacek et al., 2010 have shown increased p47 (subunit of NADPH oxidase), nitrotyrosine, MMP-9, and MMP-12 in injured arteries leading to vascular remodelling. NaSH supplementation prevented vascular remodelling by restoring normal redox stress levels and MMP levels [157]. High homocysteine concentration can disrupt the blood-brain barrier (BBB) [158]. Administration of H_2_S donors, NaSH, and N-acetyl cysteine (NAC) during pregnancy shows protective action against BBB permeability of offspring by regulating nitrite levels and proinflammatory cytokine levels [159].

Homocysteine contributes significantly to the ROS generation by mitochondria. Homocysteine activates N-methyl-D-aspartate receptor (NMDA) and induces mitochondrial toxicity by increased levels of Ca^2+^, NADPH-oxidase-4 (NOX-4) expression. NaSH treatment reduces mitochondrial oxidative stress and restores ATP, suggesting that it has protective properties against mitochondrial toxicity [160] Homocysteine induce endoplasmic reticulum (ER) stress [161]. NaSH inhibits homocysteine-mediated ER stress by upregulating silent mating type information regulator 2 homolog 1 (SIRT-1), one of the seven mammalian sirtuins, in PC12 cells [162].

SG-1002—a sodium polysulthionate—is a synthetic H_2_S prodrug that contains > 90% α-sulfur with additional trace constituents, such as sodium sulfate, sodium thiosulfate, sodium polythionate. Oral administration of SG-1002 leads to sustained release of H_2_S and sulfane-sulfur on heart failure model [163]. Hyperhomocysteinemia causes cardiac remodeling and dysfunction in vivo, which is ameliorated by slow releasing H_2_S donor SG-1002 [164]. CSE is a key enzyme involved in homocysteine metabolism; it generates H_2_S from homocysteine. Elevated homocysteine levels in the blood can downregulate CSE expression and nitrosylate CSE (CSE-SNO), reducing its catalytic activity and H_2_S production, causing atherosclerosis. GYY4137, an exogenous, long-acting H_2_S donor and NaSH upregulates CSE expression and sulfhydration (CSE-SSH), leading to decrease in homocysteine level and atheroscelerotic plaques [165] Homocysteine can upregulate mitochondrial fission marker (DRP-1), fusion marker (Mfn2), and autophagy marker (LC-3). Tetrahydrocurcumin, an anti-oxidative, anti-inflammatory compound can attenuate homocysteine mediated oxidative damage, mitochondrial fission/fusion, mitophagy [166]. Together, augmentation of cells with exogenous H_2_S can counter the effects of elevated Hcy and is a rich area for further investigation.

## Conclusion

This review summarizes different aspects of immunothrombosis in hyperhomocysteinemia condition with possible correlations between HHcy and thrombotic events in bacterial, viral and parasitic infections. The elevated homocysteine activates the immune cells such as neutrophils, monocytes, platelets and endothelial cells by various molecular mechanisms and lead to the thrombotic cascade. The relationship between homocysteine and thrombosis in line with present scientific literature is elaborated in this review with a major focus on homocysteine-induced thrombotic events in infections, with the example of COVID-19 and sepsis. Accumulating evidence from recent years has shown the therapeutic importance of targeting HHcy in addition to its diagnostic nature for various pathological conditions. It has been reported that a diet deficient in B vitamins, betaine and high methionine content is responsible for the development of HHCy. Interestingly compensation of these components must be considered of high therapeutic relevance in clinical practice. In addition to that lifestyle modifications along with a nutritional diet rich in antioxidants will decrease the severity of HHcy, being helpful in defeating the pathological conditions associated with infections. This review is intended to summarize all evidence that can show how HHcy is implicated in thrombotic events of infections mediated by immune cells to establish the therapeutic targets that can focus on the dysregulated homocysteine metabolism.

## Data Availability

No datasets were generated or analysed during the current study.
